# Arthrosen des Handgelenks

**DOI:** 10.1007/s00132-024-04502-w

**Published:** 2024-05-24

**Authors:** C. J. Deglmann

**Affiliations:** 1 MünchenHand - Privatpraxis für Hand- und Handgelenkchirurgie, Marienplatz 21, 80333 München, Deutschland; 2 Deutsches Zentrum für Obere Extremität, Effnerstr. 38, 81925 München, Deutschland

**Keywords:** Radiokarpale Arthrose, Ulnokarpale Arthrose, Konservative Maßnahmen, Operatives Verfahren, Rettungsoperationen, Radiocarpal osteoarthritis, Ulnocarpal osteoarthritis, Conservative measures, Surgical procedures, Salvage operation

## Abstract

Der Begriff Handgelenkarthrose kann als Überbegriff für verschiedene, oft unabhängige Arthrosebereiche verwendet werden, da das Handgelenk aus mehreren Teilbereichen zusammengesetzt ist. Die radiokarpale Arthrose entsteht oft nach nicht therapierten Bandverletzungen, bei fehlheilenden Knochenfrakturen im Carpus oder nach Radiusfrakturen mit Gelenkbeteiligung. Eine typische Sequenz der Ausbreitung ist bekannt für die radiokarpale Arthrose nach SL(skapholunär)-Insuffizienz oder Skaphoidpseudarthrose. Zu den weiteren Ursachen zählen Entzündungen, Kristallablagerungen oder Knochennekrosen. Die ulnokarpale Arthrose tritt bei Niveauunterschieden zwischen Ulna und Radius posttraumatisch oder auch primär auf. Bei der Therapie der Handgelenkarthrose sollte nach Ausschöpfung von konservativen Maßnahmen ein operatives Verfahren gewählt werden, das unter Berücksichtigung der operativen Risiken und des individuellen Anspruchs eine möglichst gute Belastung und Restbeweglichkeit ermöglicht. Bei Rettungsoperationen werden die defekten Knorpelbereiche entweder direkt fusioniert oder durch geeignete umleitende Teilfusionen und Resektionsarthroplastiken ausgeschaltet. Die genaue Analyse der betroffenen Zonen ist entscheidend für die Auswahl eines geeigneten Eingriffs.

## Abstract (en)

The term osteoarthritis (OA) of the wrist can be used as an umbrella term for various, often independent areas of OA, as the wrist is made up of several joints. Radiocarpal OA often occurs after untreated ligament injuries, incorrectly healed bone fractures in the carpus or after radius fractures involving the joint. A typical sequence of propagation is known for radiocarpal OA following scapholunate (SL) insufficiency or scaphoid pseudarthrosis. Other causes include inflammation, crystal deposits or bone necrosis. Ulnocarpal arthrosis occurs posttraumatically or primarily when there are differences in levels between the ulna and radius. When treating wrist arthrosis, after conservative measures have been exhausted a surgical procedure should be chosen that enables the best possible load-bearing and residual mobility, considering the surgical risks and individual requirements. During salvage operations, the defective cartilage areas are either fused directly or eliminated using appropriate diverting partial fusions and resection arthroplasty. An accurate analysis of the affected zones is crucial for selecting an appropriate intervention.

## Lernziele

Nach Lektüre dieses Beitrags …

- haben Sie grundlegende Kenntnisse über die Untersuchung des Handgelenks mit seinen Teilgelenken,
- ist es Ihnen möglich, die häufigsten Arthrosearten am Handgelenk klinisch zu unterscheiden,
- kennen Sie die Pathogenese und Stadien der radiokarpalen Arthrose nach SL(skapholunär)-Band Läsion (SLAC[„scapholunate advanced collapse“]-Wrist) und Skaphoidpseudarthrose (SNAC[„scaphoid nonunion advanced collapse“]-Wrist),
- haben Sie einen Überblick über therapeutische Alternativen bei Arthrose am Handgelenk,
- sind Ihnen die wichtigsten Rettungsoperationen bei aufgetretener Arthrose radiokarpal und radioulnar bekannt.

## Einleitung

Die **radiologische Prävalenz**

radiologische Prävalenz

einer Handgelenkarthrose wird bei einer insgesamt limitierten und heterogenen Studienlage in der Framingham-Studie mittels ausschließlich radiologischer Diagnosestellung mit 1–2 % angegeben – deutlich seltener als andere Arthrosen im Bereich der Hand [1]. Das Vorhandensein radiologischer Arthrosezeichen erscheint allerdings in Studien möglicherweise häufiger als die tatsächliche subjektive Angabe von Beschwerden [2]. Eine primäre Arthrose am Handgelenk ist vergleichsweise selten. Nur bei der isolierten skaphotrapezotrapezoidalen (STT) Arthrose findet sich häufig keine erkennbare Prädisposition.

Eine **radiokarpale Handgelenkarthrose**

radiokarpale Handgelenkarthrose

kann bei entzündlichen Erkrankungen, Kristallablagerungen, unbehandelten Knochennekrosen wie der Lunatum- oder der Skaphoidnekrose auftreten, die wieder potenziell auch eine posttraumatische Genese haben könnten [3].

**Posttraumatische Knorpelläsionen**

Posttraumatische Knorpelläsionen

können nach Bandläsionen am Carpus oder nach Frakturen am Carpus und am Radius auftreten. Eine bekannte Progression der SLAC(„scapholunate advanced collapse“)-Wrist nach SL-Bandverletzung und der vorhersehbaren Ausbreitung der radiokarpalen Arthrose bis nach mediokarpal wurde bereits früh von Watson und Ballet beschrieben [4]. Ähnliche Verläufe treten nach instabiler Skaphoidpseudarthrose als SNAC(„scaphoid nonunion advanced collapse“)-Wrist auf [5].

Bei **angeborenen Fehlstellungen**

angeborenen Fehlstellungen

wie der Madelung-Deformität können atypische anatomische Veränderungen radiokarpal, ulnokarpal oder radioulnar zu Knorpelläsionen führen [6]. Bei der ulnokarpalen Arthrose ist häufig eine primäre oder sekundäre Ulnaplusvariante bei der Entstehung ursächlich.

## Anamnese und klinische Untersuchung

Für die Planung einer Therapie sind eine genaue **Anamneseerhebung**

Anamneseerhebung

und klinische Untersuchung entscheidend. Die Anamnese sollte vorausgegangene Traumen, entzündliche Systemerkrankungen, den zeitlichen Verlauf der Beschwerden und die Tagesdynamik einschließen. Zudem soll evaluiert werden, ob die Beschwerden radial, ulnar oder zentral auftreten und bei welchen Gelegenheiten. Sinnvoll kann die Verwendung einer visuellen Analogskala (VAS) bei der Beurteilung von Belastungs- und Ruheschmerzen sein.

Die **klinische Untersuchung**

klinische Untersuchung

der aktiven Extension und Flexion sowie der Pro- und Supination gehören zur **Dokumentation**

Dokumentation.

Die Palpation radiokarpal, zentral dorsal und ulnokarpal hilft bei der anatomischen Eingrenzung. Die Extension mit leicht forciertem Druck nach dorsal, der Watson-Test, die Stabilität des distalen radioulnaren Gelenks (DRUG) in Pro- und Supination, die Kompression des DRUGs und die Palpation des TFCCs (triangulärer fibrokartilaginärer Komplex) erlauben weitere detaillierte Eingrenzungen [7, 8]. Weiterhin ist die Untersuchung der radialen und ulnaren, beugeseitigen und streckseitigen Sehnen zur Beurteilung einer reaktiven oder primären Tendinopathie sinnvoll, insbesondere zur Abgrenzung gegen intraartikuläre Pathologien. Wichtig ist die Untersuchung der ECU(Extensor carpi ulnaris)-Sehne als einzige mit der Ulna ziehende Sehne, die bei ulnokarpalen Pathologien begleitend entzündet sein kann. Mit dem **„ECU synergy test“**

„ECU synergy test“

kann durch radiale Daumenabduktion gegen Widerstand eine Tendinitis der ECU-Sehne gezeigt werden. Der **reverse Finkelstein-Test**

reverse Finkelstein-Test

erzeugt Schmerzen an der ECU-Sehne bei passiver Beugung und Radialduktion im Handgelenk [9, 10].

## Bildgebende Diagnostik

**Figure 1:**
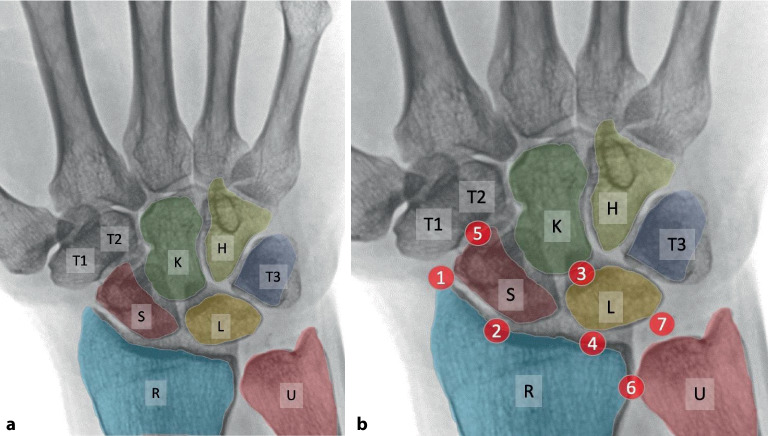

**Figure 2:**
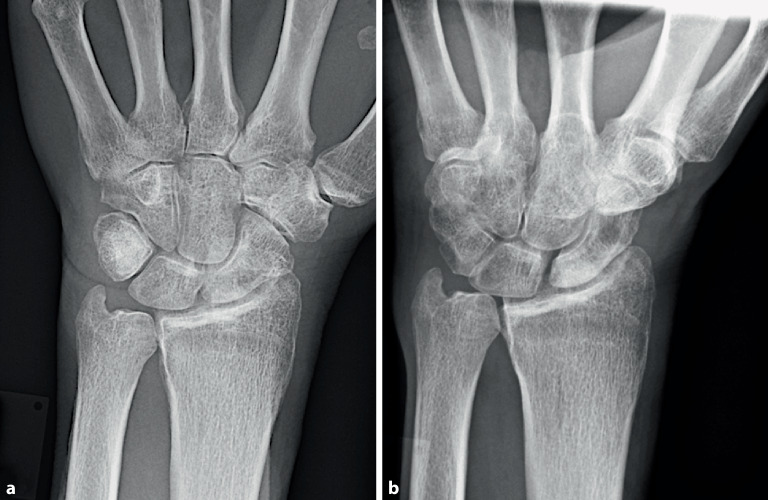

Eine **konventionelle Röntgenaufnahme**

konventionelle Röntgenaufnahme

(Projektionsradiographie) des Handgelenks in 2 Ebenen ist eine gute Basisdiagnostik für die Handgelenkarthrose (Abb. 1). Die **dorsopalmare Aufnahme**

dorsopalmare Aufnahme

sollte in Oberarmelevation und -abduktion und Ellenbeugung von 90° in der Neutralstellung erfolgen. Bei der **seitlichen Aufnahme**

seitlichen Aufnahme

soll die Schulter adduziert und das Ellenbogengelenk 90° gebeugt sein. Bei ulnokarpalen Beschwerden ist zur Detektion einer dynamischen Ulnaplusvariante zusätzlich die Durchführung einer **dorsopalmaren Faustschlussaufnahme**

dorsopalmaren Faustschlussaufnahme

(mit forciertem Faustschluss, ggf. unter Zuhilfenahme eines Balls) sinnvoll ([11]; Abb. 2).

**Table 1:** 

Therapieoptionen	Denervierung	Radiale Styloidektomie	PRC	MKTA	STT-Fusion	STT-RSA	RSL-Fusion	ASK-Wafer	Ulnakopfprothese
*1* SLAC I	X	X	–	–	–	–	–	–	–
*1 2* SLAC II	X	–	X	X	–	–	–	–	–
*1 2 3* SLAC III	X	–	–	X	–	–	–	–	–
*1* SNAC I	X	X	–	–	–	–	–	–	–
*1 2* SNAC II	X	–	X	X	–	–	–	–	–
*1 2 3* SNAC III	X	–	–	X	–	–	–	–	–
*2 4* RC-Arthrose nach DRF	X	–	–	–	–	–	X	–	–
*3* MC-Arthrose	X	–	–	X	–	–	–	–	–
*5* STT-Arthrose	–	–	–	–	X	X	–	–	–
*6* DRUG-Arthrose	X	–	–	–	–	–	–	–	X
*7* Ulnaimpaktion	–	–	–	–	–	–	–	X	–

Anatomische Korrelation mit Abb. 1b (rot umrandete Zahlen)

*PRC* proximale Reihenresektion, *MKTA* mediokarpale Teilarthrodese, *STT-Fusion* Skaphotrapeziotrapezoid-Arthrodese, *STT-RSA* Skaphotrapeziotrapezoid-Resektionsarthroplastik (arthroskopisch oder offen), *RSL-Fusion* radioskapholunäre Fusion, *ASK-Wafer* arthroskopische Teilresektion distaler Ulnakopf, *DRF* distale Radiusfraktur, *RC* radiokarpal, *MC* mediokarpal, *SLAC* „scapholunate advanced collapse“, *SNAC* „scaphoid nonunion advanced collapse“

Insbesondere bei ulnokarpalen Beschwerden und zur Abklärung von TFCC-Läsionen ist die Durchführung einer **Magnetresonanztomographie**

Magnetresonanztomographie

(MRT) sinnvoll [12, 13], auch wenn die **Arthroskopie**

**Figure 3:**
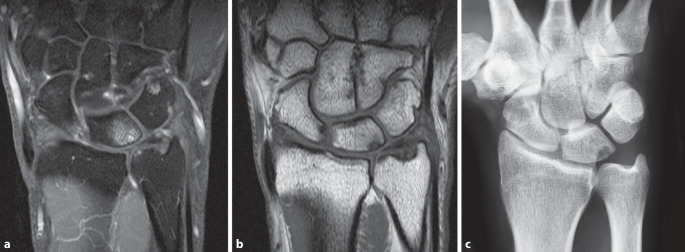

Arthroskopie

den Goldstandard für die TFCC-Beurteilung darstellt. Die Verwendung eines hochauflösenden MRT-Gerätes mit einer dezidierten Spule ist wünschenswert. Beurteilt werden der TFCC, mögliche Bandverletzungen, Knorpelbeschaffenheit und reaktive Begleitsynovitis. Bei **ulnokarpalen Impaktionssyndromen**

ulnokarpalen Impaktionssyndromen

finden sich Knochenödeme, häufig ulnarseitig proximal am Os lunatum mit zentralen Diskusläsionen oder Ausdünnungen (Abb. 3). Begleitend kann die Hamatumspitze bei Vorliegen einer besonderen Form des Lunatums (Typ 2 nach Viegas) akzentuiert dargestellt sein als Hinweis auf ein Hamatumspitzensyndrom. Ein weiteres Augenmerk sollte auf eine ECU-Tendinitis als Zeichen einer reaktiven Tenosynovitis bei ulnokarpalen Pathologien gelegt werden. Eine Tenosynovitis stellt sich mit einem ringförmigen Halo um die Sehne, degenerativen oder entzündlichen Substanzdefekten mit erhöhtem Signal dar. Subluxationen der ECU-Sehne aus ihrer Knochenrinne an der Ulna, in der sie durch eine fibröse Membran („subsheath“) gehalten wird, können einfach in den axialen Schichten diagnostiziert werden. Auch reaktive sekundäre Überlastungsreaktionen im 1. und 2. Streckerfach können bei ulnokarpalen Impaktionssyndromen beobachtet werden. Bei einer Knochennekrose, wie der Lunatumnekrose kann in der **MRT mit Kontrastmittel**

MRT mit Kontrastmittel

eine genauere Beurteilung der knöchernen Perfusion erfolgen.

Im **Ultraschall**

**Figure 4:**
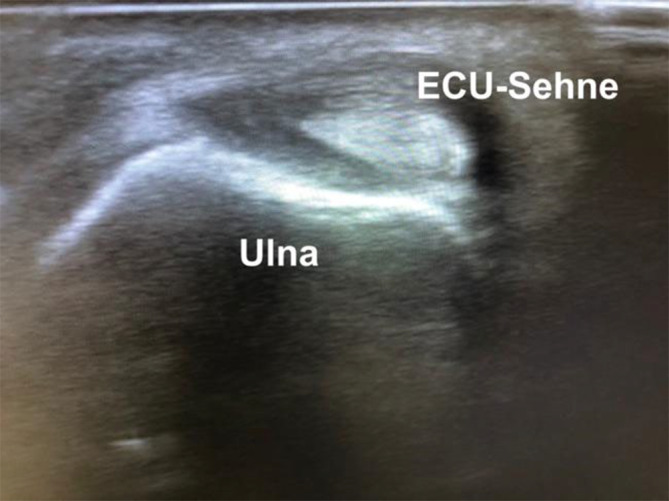

Ultraschall

können diese Sehnenscheidenentzündungen ebenfalls nachgewiesen werden (Abb. 4). Ebenso kann eine Luxationstendenz der ECU-Sehne dynamisch unter Pro- und Supination untersucht werden.

Bei einer Instabilität im DRUG ist eine CT(Computertomographie)-Untersuchung in Pro- und Supination empfohlen, wobei diese bei der fortgeschrittenen Arthrose vermutlich entbehrlich ist [12, 14].

Bei einer Ulnaimpaktion kommt es adaptiv häufiger zu einer gewissen Instabilität und Dorsalstellung der Ulna. Wird dann der TFCC, der sich zuvor adaptiv foveal gelockert hatte, refixiert, wird die Ulnaimpaktion erneut forciert, da die Ulna wieder zum Os lunatum gezogen wird.

Vor einer operativen Therapie bei einer radiokarpalen Arthrose kann die Diagnostik sinnvollerweise durch eine **Computertomographie**

Computertomographie

(CT) oder eine strahlungsärmere **digitale Volumentomographie**

digitale Volumentomographie

(DVT) ergänzt werden, um präzise Information über die Gelenkbeschaffenheit im Bereich der Fossa lunata, der Fossa scaphoidea sowie des mediokarpalen Gelenkspaltes inklusive des STT-Gelenks zu bekommen. Diese Informationen werden zur Auswahl möglicher Rettungsoperationen benötigt [6, 15]. Sollte keine ausreichende Beurteilung mit bildgebender Diagnostik erreicht werden, kann in ausgewählten Fällen eine **arthroskopische Klärung**

arthroskopische Klärung

notwendig werden, üblicherweise direkt mit der zielführenden operativen Therapie zusammen.

## Radiokarpale Arthrose

Der radiokarpale Gelenkanteil überträgt die Hauptlast der axialen Kraftwirkung zum Unterarm, was eine Arthrosebildung begünstigt. Die beiden konkaven Gelenkflächen der Fossa scaphoidea und der Fossa lunata artikulieren mit den korrespondierenden Gelenkflächen des Os scaphoideum und des Os lunatum und somit mit dem Carpus. Der Carpus besteht aus mehreren Handwurzelknochen, die miteinander in einer komplexen spiralförmigen ligamentären Verbindung stehen [16].

**Degenerative Veränderungen**

Degenerative Veränderungen

in dieser Hauptbelastungszone des Handgelenks bilden sich bei Fehlstellungen nach Bandverletzungen des Carpus oder nach nicht verheilter Skaphoidfraktur in einer typischen Sequenz von radial beginnend aus und setzen sich nach mediokarpal fort (SLAC-Wrist, SNAC-Wrist) [5]. Diese Sequenz wird noch gesondert beschrieben.

Bei der **Chondrokalzinose**

**Figure 5:**
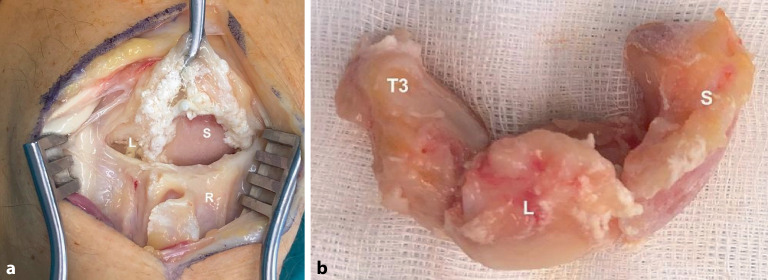

Chondrokalzinose

(„calcium pyrophosphate deposition disease“ [CPPD]) kommt es zu kristallinen Ablagen an den interkarpalen Bändern mit Bandrupturen in Folge, was die SLAC-Sequenz auslöst (Abb. 5). Diese Sequenz kann auch als SCAC(„scaphoid chondrocalcinosis advanced collapse“)-Wrist genannt. Zudem finden sich die kristallinen Ablagerungen am TFCC und im STT-Gelenk mit einer STT-Arthrose in Folge [17]. Gichtarthropathien am Handgelenk sind dagegen eher selten.

Ein weiterer Grund für eine arthrotische Veränderung radiokarpal ist die fortgeschrittene **Lunatumnekrose**

Lunatumnekrose.

Bei der schwer verlaufenden Mondbeinnekrose kommt es zur Höhenminderung und meist sagittalen Infraktion des Os lunatum mit der Folge eines karpalen Kollapses. Diese kann im Verlauf zu einer degenerativen Veränderung im Bereich der Fossa lunata und später auch zu degenerativen Veränderungen mediokarpal führen [18].

Nach **intraartikulären Radiusfrakturen**

intraartikulären Radiusfrakturen

mit ungenügender anatomischer Rekonstruktion der Gelenkfläche, insbesondere bei verbleibenden Gelenkstufen, kann es zu progredienten Knorpelläsionen auch der gegenüberliegenden Knorpelflächen radiokarpal und einer fortschreitenden Arthrose kommen [15]. Heilt die Radiuskonsole in Fehlstellung ab, kommt es zu adaptiven Veränderungen im Carpus mit Einschränkung der Beweglichkeit, potenziell Schmerzen und im weiteren Verlauf auch zu einer Arthrose. Diese sekundären Veränderungen können in die Gruppe der CIA („carpal instability adaptive“) eingeteilt werden als besondere Untergruppe der Instabilitäten, die initial bei der Mayo-Klassifikation der karpalen nichtdissoziativen Instabilitäten (CIND) noch ausgeschlossen war [19, 20, 21, 22].

**Entzündliche Erkrankungen**

Entzündliche Erkrankungen

können ebenfalls ein Mischbild der Degeneration erzeugen, die aufgrund von Bandinsuffizienzen und direkten Knorpelläsionen entstehen, oft allerdings ulnokarpal beginnend und oft über das Handgelenk hinausreichend [23]. Auch wenn Überschneidungen bestehen, wird in dieser Ausführung nicht auf die Arthritis eingegangen.

### Sequenz: radiokarpale Arthrose nach karpalen Instabilitäten (SLAC-, SNAC-, SCAC-Wrist)

Das **Os scaphoideum**

Os scaphoideum

zeigt eine komplexe Beweglichkeit bei Extension und Flexion sowie Radial- und Ulnarduktion des Handgelenks. Bekannt ist auch die sog. „dart throwing motion“ (Dart-Wurfbewegung), die aus der mediokarpalen Gelenkreihe heraus entsteht. Dabei besteht eine komplexe Zusammenarbeit von intrinsischen und extrinsischen Bändern am Carpus und radiokarpal [16].

Ist die Stabilisierung des Skaphoids verändert, wird durch den palmaren Druck des Os trapezium und des Os trapezoideum das palmar flektierte Os scaphoideum auf die dorsale Lippe des Radius gedrückt und erzeugt durch eine punktförmige Belastung von radial her im Verlauf eine Knorpelläsion, die sich dann weiter über die Fossa scaphoidea ausbreitet.

Die Sequenzen sind ähnlich, die Genese der **Skaphoidfehlstellung**

Skaphoidfehlstellung

unterschiedlich: Bei der **SLAC-Wrist**

SLAC-Wrist

ist der Auslöser eine Insuffizienz im SL-Band-Komplex und die Entkoppelung vom Mondbein. Diese Pathologie kann auch unter Überbegriff CID („carpal instability dissociative“) subsumiert werden. Dieser Begriff beschreibt eine Instabilität zwischen 2 karpalen Knochen in einer karpalen Reihe, während die CIND („carpal instability non dissociative“) eine Instabilität der gesamten proximalen Reihe gegenüber dem Radius oder/und der distalen Reihe beschreibt [19, 21]. Das Os scaphoideum geht bei der Entkoppelung zunehmend in eine flektierte Stellung, das Os lunatum in eine extendierte Stellung, hier DISI(„dorsally intercalated segment instability“)-Stellung genannt. Diese grundlegende Beobachtung wurde bereits von Watson und Ballet 1984 beschrieben [4]. Zunächst ist der radiale Anteil von einem Knorpelschaden betroffen (Stadium 1), dann die gesamte Fossa scaphoidea (Stadium 2) und später auch der mediokarpale Gelenkanteil zwischen dem Os capitatum und dem Os lunatum (Stadium 3) beteiligt [5]. Eine Panarthrose im Handgelenk wird gelegentlich auch als 4. Stadium beschrieben, wobei hier häufig andere Kofaktoren beitragen [15].

Nach einer instabilen Pseudarthrose am Skaphoid kommt es durch die palmare Abkippung des distalen Skaphoidfragments radial an der Fossa scaphoidea zu der Folge einer **SNAC-Wrist**

SNAC-Wrist.

Dabei wird das distale Skaphoidfragment nach dorsal gedrückt, und wie bei der SLAC-Wrist beginnt die Arthrose radial (Stadium 1). Im Verlauf setzt sich die Knorpelläsion in der Fossa scaphoidea bis zum Pseudarthrosenspalt (Stadium 2) und dem proximalen Pol, der noch mit dem Os lunatum verbunden ist, fort und später auch mediokarpal (Stadium 3) [5].

Bei der SLAC-, SNAC- und **SCAC-Wrist**

SCAC-Wrist

bleibt die Fossa lunata für längere Zeit intakt. Daher sind die Beurteilung der Gelenkflächen an der Fossa lunata und dem korrespondierendem Os lunatum als auch die Beurteilung der Gelenkfläche zwischen Os lunatum und Os capitatum mediokarpal für die weitere Therapieentscheidung und Auswahl einer geeigneten Rettungsoperation wichtig. Im Zweifel kann eine begleitende Arthroskopie bei einer Rettungsoperation noch diagnostische Klarheit schaffen.

## Mediokarpale Arthrose und skaphotrapezotrapezoidale Arthrose

Die **mediokarpale Arthrose**

mediokarpale Arthrose

ist meist die tertiäre Form bei der SLAC, SNAC- oder SCAC-Wrist und kommt dann zusammen mit einer radiokarpalen Arthrose vor.

Eine Arthrosebildung zwischen dem Os lunatum und dem Os capitatum ist isoliert möglich, aber selten. Auslöser kann beispielsweise eine mediokarpale Instabilität mit Luxationstendenz mediokarpal sein [24].

Eine seltenere mögliche mediokarpale Arthroselokalisation ist die Spitze des Os hamatum. Alleine oder verstärkt bei einer Ulnaimpaktion kann es zu einer Impaktion der **Hamatumspitze**

Hamatumspitze

am gegenüberliegenden Teil des Os lunatum bei Vorliegen eines Os lunatum vom Typ II kommen [25]. Eine vermehrte Kontrastaufnahme an der Hamatumspitze ist in der MRT hinweisend auf ein Hamatumspitzensyndrom. Die Diagnose wird arthroskopisch gestellt.

Die STT-Arthrose tritt zwischen dem Os scaphoideum, dem Os trapezium und dem Os trapezoideum auf. Die Genese kann primär, aber auch sekundär bei mediokarpalen Instabilitäten sein. Eine Chondrokalzinose (CPPD) wurde als auslösender Zustand bereits beschrieben.

## Radioulnokarpale Handgelenkarthrose

Es gibt verschiedene anatomische Möglichkeiten, den ulnaren Gelenkbereich zu unterteilen. Funktionell ist es sinnvoll, den radioulnokarpalen Bereich zusammenzufassen, der aus dem distalen radioulnaren Gelenk (DRUG) und dem ulnokarpalen Gelenkbereich besteht [26].

Ulnokarpal (zwischen der Ulna und dem Carpus mit dem zwischengelagerten Puffer- und Stabilitätskomplex des TFCC) werden in etwa 10–20 % der Kraft übertragen. Dabei ist die tatsächliche Belastung abhängig von der Ulnalänge und der Handgelenkstellung [13, 27, 28].

**Degenerative Veränderungen**

Degenerative Veränderungen

im ulnokarpalen Bereich stehen häufig im Zusammenhang mit einem Niveauunterschied zwischen dem distalen Radius und der distalen Ulna. Der funktionelle **Längenunterschied**

Längenunterschied

kann angeboren, sekundär z. B. nach eingestauchten Radiusfrakturen, überschießendem Wachstum nach kindlichen Unterarmfrakturen oder auch bei altersbedingter Höhenabnahme der radiokarpalen Knorpelhöhe auftreten. Der Unterschied kann statisch sein und bereits auf der d.p.(dorsopalmar)-Röntgenübersichtsaufnahme erkennbar sein oder dynamisch. In diesem Fall sind die Stabilisatoren zwischen Ulna und Radius laxer, was bei einer Belastungssituation den ulnokarpalen Gelenkraum verschmälert [11]. Bei einer längeren Ulna kommt der TFCC-Komplex unter erhöhten Druck. Dies kann zunächst zu degenerativen zentralen TFCC-Läsionen, später dann zu Knorpelläsionen an den korrespondierenden Gelenkflächen am Caput ulnae und dem ulnaren Os lunatum führen [11, 13, 29].

Klinische Zeichen für die **Ulnaimpaktion**

Ulnaimpaktion

sind ulnokarpale belastungsabhängige Beschwerden, die beim Abstützen auf das extendierte Handgelenk, bei belasteter Ulnarduktion oder Umwendungsbewegungen unter Belastung des Handgelenks auftreten.

Insuffizienzen im Bereich des distalen radioulnaren Gelenks können zu einer **DRUG-Arthrose**

DRUG-Arthrose

führen. Die Stabilisierung des DRUG wird durch mehrere anatomische Strukturen aktiv und passiv gewährleistet, darunter der TFCC und die radioulnaren Bänder [11, 12, 13]. Zudem können auch Radiusfrakturen, die in den DRUG-Spalt einziehen und nicht anatomisch rekonstruiert wurden, zu einer Knorpelläsion führen. Oft wird auch die zugrunde liegende Pathologie erst bemerkt, wenn bereits ein Knorpelschaden vorliegt.

Bei **angeborenen Fehlstellungen**

angeborenen Fehlstellungen

wie der Madelung-Deformität oder der Madelung-like-Deformität führen anatomische Veränderungen an der mediopalmaren Radiusmetaphyse radiokarpal zu Überlastungen, ulnokarpal zu einer Ulnaimpaktion und/oder radioulnar durch eine Fehlstellung im DRUG zu degenerativen Veränderungen [6]. Es treten auch abgeschwächte Formen auf, die sich nicht relevant von einem Ulnaimpaktionssyndrom unterscheiden.

## Therapie der Handgelenkarthrose

### Handgelenkdenervierung, arthroskopische Synovektomie

Bei der Therapie sind, wenn noch vertretbar, **konservative Therapiemaßnahmen**

konservative Therapiemaßnahmen

voranzustellen. Eine qualifizierte Handtherapie, die insbesondere auch die Propriozeption trainiert und eine Gelenkschulung einschließt, wird empfohlen [30]. Bei einer präarthrotischen Situation soll die zugrunde liegende Pathologie adressiert werden. Außerdem ist das Fortschreiten der SLAC/SNAC-Sequenz zu beachten, da bei jedem weiteren Schweregrad mögliche bewegungserhaltende Therapien wegfallen.

Als niederschwellige therapeutische Maßnahme kann insbesondere bei höhergradigen radiokarpalen Arthrosen eine **operative Denervierung des Handgelenks**

operative Denervierung des Handgelenks

diskutiert werden [31, 32]. Die Effektivität ist nicht komplett vorhersehbar, zudem wird die Rolle einer möglicherweise reduzierten Propriozeption leidenschaftlich diskutiert. Dies machte den Eingriff zeitweise unpopulär. Es gibt zudem verschiedene Varianten, von einer kleinen Inzision mit Resektion des N. interosseus posterior und des N. interosseus anterior bis zu einer kompletten Denervation, die mehrere Inzisionen benötigt. Eine vorgeschaltete **Probedenervierung**

Probedenervierung

mit Lokalanästhetika verbessert die Prädiktion: Dabei werden die wichtigsten Zielpunkte der späteren Denervierung mit einem Lokalanästhetikum infiltriert. In der Wirkungszeit der lokalen Anästhesie soll der Patient eine Belastungserprobung durchführen und die Ergebnisse selbst dokumentieren. Werden die Beschwerden bei der Probedenervierung gut gebessert, ist eine operative Denervierung zu erwägen. Diese kann in WALANT („wide awake local anesthesia no tourniquet“) als kleiner Eingriff ohne Ruhigstellung und ohne Einschränkung der ROM („range of motion“) erfolgen und ist insbesondere dann empfohlen, wenn keine größere Belastung erfolgen muss oder wenn aus persönlichen oder medizinischen Gründen kein größerer Eingriff möglich oder gewünscht ist. In einigen Studien wurde auch nach mehreren Jahren eine deutliche symptomatische Verbesserung beschrieben [31, 32, 33].

Eine **arthroskopische Synovektomie**

arthroskopische Synovektomie,

ggf. auch mit arthroskopischer Styloidektomie und mit einer Denervierung, kann auch bei höhergradigen Arthrosen am Handgelenk symptomatisch begleitend durchgeführt werden, wobei hier größere Fallserien fehlen [34].

### Arthroskopische Resektionen mediokarpal

Liegt eine STT-Arthrose simultan mit einer Rhizarthrose vor, ist eine offene **Resektionsarthroplastik**

**Figure 6:**
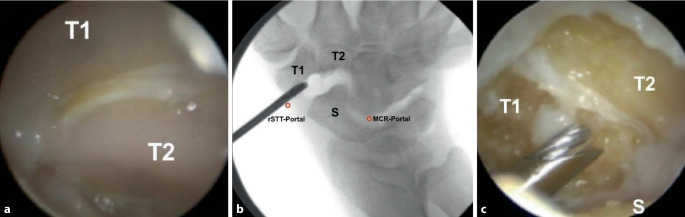

Resektionsarthroplastik

mit Trapezektomie und ggf. proximaler Teiltrapezoidektomie eine zuverlässige Therapieoption [35]. Bei einer isolierten STT-Arthrose hebt eine arthroskopische Resektionsarthroplastik den Knochenkontakt zwischen Os trapezium, Os trapezoideum und Os scaphoideum auf. Arthroskopische Resektionen erfordern arthroskopische Erfahrung ([36]; Abb. 6).

Bei einem **Hamatumspitzensyndrom**

Hamatumspitzensyndrom

kann arthroskopisch die Hamatumspitze reseziert werden [25].

### Therapie bei SLAC-/SNAC-Wrist-Stadium I

Bei einer erstgradigen radialseitigen Knorpelläsion (SLAC-/SNAC-Stadium I) ist die Resektion des **Processus styloideus radii**

Processus styloideus radii

(PSR), vorzugsweise arthroskopisch, eine therapeutische Option. Um ein Fortschreiten der Arthrose zu verhindern, sollte der Grund der karpalen Instabilität im gleichen Schritt korrigiert, also eine SL-Bandläsion therapiert oder eine Skaphoidpseudarthrose rekonstruiert werden [15].

### Rettungsoperationen

Sind die Beschwerden länger anhaltend und ist eine größere Belastung im Alltag notwendig, kommen sog. „Rettungsoperationen“ als Therapieoption infrage. Bei diesen Operationen wird jeweils die Belastungszone durch Resektion oder Fusionen umgeleitet auf die verbliebenen intakten Gelenkteile. Die **operative Zielsetzung**

operative Zielsetzung

ist die größtmögliche Beweglichkeit und Belastbarkeit bei möglichst geringem Risiko.

Häufige Rettungsoperation sind die mediokarpale Teilarthrodese, die proximale Reihenresektion, die STT-Arthrodese und die RSL(radioskapholunär)-Fusion.

#### Mediokarpale Teilarthrodese

Bei der mediokarpalen Teilarthrodese (MKTA) oder **Four-Corner-Fusion**

Four-Corner-Fusion

(4CF) wird das Os scaphoideum reseziert und das Os lunatum mit dem Os capitatum und das Os triquetrum mit dem Os hamatum fusioniert.

Die Fossa scaphoidea und das STT-Gelenk werden ausgeschaltet, mediokarpal wird fusioniert. Daher kann der Eingriff für SLAC/SNAC-Wrist-Stadium II und III oder anderen Arthroseformen mit intakter Fossa lunata eingesetzt werden. Wichtige Voraussetzung ist eine stabile ligamentäre Verbindung des Os lunatum mit dem Radius.

Die **Osteosynthese**

Osteosynthese

wird entweder mit Kirschner-Drähten, kanülierten Schrauben oder auch speziellen Platten durchgeführt, wobei typischerweise Radiusspongiosa und Spongiosa vom resezierten Os scaphoideum interponiert werden. Gelingt es, das Os lunatum aus der oft bestehenden DISI-Fehlstellung zu bringen, kann eine Beweglichkeit von ca. 30° Beugung und 30° Streckung des Handgelenks erwartet werden, häufig auch mehr. Die Kraft wird nun v. a. über die Säule Os capitatum und Os lunatum übertragen (siehe klinisches Beispiel Abb. 11). Es gibt einige Varianten der Fusionen, klinisch sind die Ergebnisse aber in der Regel vergleichbar [15, 37, 38].

#### Proximale Reihenresektion

Die proximale Reihenresektion („proximal row carpectomy“ [PRC]) ist eine reine Resektionsarthroplastik. Das Os scaphoideum, das Os lunatum und das Os triquetrum werden entfernt. Das Os capitatum senkt sich in die Fossa lunata und bildet ein **Neogelenk**

Neogelenk.

Daher ist die knorpelige Intaktheit am Os capitatum und an der Fossa lunata des Radius eine zwingende Voraussetzung. Damit scheidet die SLAC-/SNAC-Wrist im Stadium III aus, da mediokarpal bereits ein Knorpelschaden eingetreten ist. SLAC/SNAC im Stadium II sind typische Indikationen wie auch die Lunatumnekrose (siehe klinisches Beispiel Abb. 10).

Einige mögliche begleitend bestehende Probleme löst die PRC automatisch: Das STT-Gelenk wird aufgelöst, eine Ulnaimpaktion bessert sich meist. Die PRC erfordert keine ossäre Knochenheilung, keine Osteosynthese und ist daher auch gut für ältere Patienten geeignet. Sie zeichnet sich durch ein **niedrigeres Risikoprofil**

niedrigeres Risikoprofil

für perioperative Komplikationen verglichen mit der MKTA aus. Kurzfristig ist die Grobkraft reduziert, da die tendinöse Vorspannung aufgrund der Carpushöhenreduktion reduziert wird. Langfristig ist die PRC vergleichbar mit der mediokarpalen Teilarthrodese. Eine Arthrosebildung im Neogelenk zwischen dem Os capitatum und dem Radius ist möglich, weshalb bei jungen Patienten mit hohem Belastungswunsch oftmals eher zur mediokarpalen Teilarthrodese geraten wird – auch vom Autor – trotz der fehlenden Unterstützung für dieses Vorgehen in der Literatur. Die Wahl zwischen einer MKTA und PRC ist vermutlich oft von einem Bias des Behandlers geprägt. Positiv formuliert ist eine individuelle Beratung des Patienten sinnvoll. Eine aROM (active Range of Motion) von ca. 70–80° kann erwartet werden bei einer PRC [39, 40, 41].

#### Skaphotrapeziotrapezoid-Arthrodese

Bei der **STT-Fusion**

STT-Fusion

wird das Os scaphoideum mit dem Os trapezium und dem OS trapezoideum fusioniert. Die Kraftübertragung wird vermehrt auf die Fossa scaphoidea gelegt, das Lunatum wird entlastet und das STT-Gelenk ausgeschaltet. Diese Fusion verändert die Biomechanik des Os scaphoideum in der Fossa scaphoidea und kann dadurch im Verlauf zu einer degenerativen Veränderung in der Fossa scaphoidea führen. Der Eingriff wird mit einem sehr variablen **Pseudarthrosenrisiko**

Pseudarthrosenrisiko

von 3,5 % bis über 20 % angegeben [37, 42]. Die Verwendung von Radiusspongiosa oder besser eines Beckenkammspans, der anatomischen Einstellung des Skaphoidwinkels und einer stabilen Osteosynthese z. B. mit kanülierten versenkbaren Schrauben zeigt erfahrungsgemäß eine zuverlässigere Konsolidierung. Eine vorsichtige Resektion des radialen Styloids wird teilweise empfohlen. Bei der isolierten STT-Arthrose wird dieser Eingriff nicht mehr häufig eingesetzt, da die Resektionsarthroplastiken arthroskopisch oder offen in der Regel verlässliche Ergebnisse bei niedrigeren Komplikationsraten bringen [35]. Da die STT-Arthrodese eine spätere Umwandlung in eine PRC immer noch ermöglicht, ist sie eine verbleibende Option bei Patienten mit einer höhergradigen Lunatumnekrose (mindestens Stadium Lichtmann IIIB), die in diesem Beitrag nur kurz erwähnt wird [43].

#### Radioskapholunäre Fusion

Die RSL-Fusion reduziert die Bewegung auf die mediokarpale Gelenkreihe. Das Os scaphoideum und das Os lunatum werden mit dem Radius fusioniert. Das Radiokarpalgelenk wird damit komplett ausgeschaltet, was nach fehlverheilten intraartikulären Radiusfrakturen mit Destruktion der Fossa scaphoidea und der Fossa lunata indiziert ist. Durch die Resektion des distalen Skaphoids kann dabei die **Beweglichkeit**

Beweglichkeit

nochmals deutlich verbessert und die Pseudarthrosenrate reduziert werden [44]. Die Arthrodese kann mit Drähten, kanülierten Schrauben oder auch Platten durchgeführt werden, die Interposition eines Beckenkammspans ermöglicht eine ausreichende Höhe des Carpus zur Vermeidung einer Ulnaimpaktion. Dabei kann von palmar oder von dorsal aus vorgegangen werden, wobei oft palmar ohnehin eine Platte vom Radius entfernt werden muss und von hier aus gleich fusioniert werden kann. Eine aROM von mindestens 50° kann bei guter Grobkraft erwartet werden [45, 46].

#### Panarthrodese

Die **vollständige Handgelenkarthrodese**

vollständige Handgelenkarthrodese

wird nicht mehr so häufig eingesetzt. Dabei kann eine komplette Destruktion radiokarpal als auch mediokarpal überbrückt werden. Die Fusion kann auch nach einer proximalen Reihenresektion bei Ausbildung einer radiokapitalen Arthrose als „last resort“ erfolgen. Die Arthrodese schloss früher das **CMC-Gelenk**

CMC-Gelenk

(Karpometakarpalgelenk) mit ein, heute wird häufiger eine radiokarpale Panarthrodese ohne CMC-Einschluss nach Interposition eines Beckenkammspans mit speziellen winkelstabilen Platten von dorsal durchgeführt. Die geplante Stellung der Arthrodese kann präoperativ mit einer Orthese simuliert für den Patienten und Anpassungen an den Alltag optimiert werden. Die Extension und die Flexion sind aufgehoben, die Umwendung im DRUG ist weiterhin möglich [37, 47].

### Therapie bei radioulnokarpaler Arthrose

#### Therapie der Ulnaimpaktion

Die operative Therapie bei der Ulnaimpaktion zielt auf die relative **Verkürzung der Ulna**

Verkürzung der Ulna

und die damit verbundene Reduktion der Kraftübertragung ulnokarpal. Ist eine Fehlstellung des distalen Radius wie bei der Madelung-Deformität oder nach Radiusfrakturen ursächlich, kann eine Korrekturosteotomie des distalen Radius erfolgen. Wesentlich häufiger wird bei regelrechter Stellung des distalen Radius die Durchführung einer Arthroskopie des Handgelenks mit Synovektomie, TFCC-Débridement und ggf. Diskusteilresektion erfolgen, was häufig bereits eine deutliche Verbesserung erbringt. Ferner kann – entweder sekundär oder bereits simultan bei hochgradigen ulnokarpalen Arthrosezeichen – eine offene Ulnaverkürzung oder eine arthroskopische intraartikuläre UInaverkürzung (Wafer-Procedure) erfolgen [48]. Eine **Wafer-Procedure**

Wafer-Procedure

ist dann gut durchführbar, wenn der TFCC bereits zentral perforiert ist, was bei den hochgradigen Arthrose-induzierenden Impaktionen in der Regel der Fall ist. Anderenfalls müsste eine iatrogene Perforation des TFCC erfolgen.

**Figure 7:**
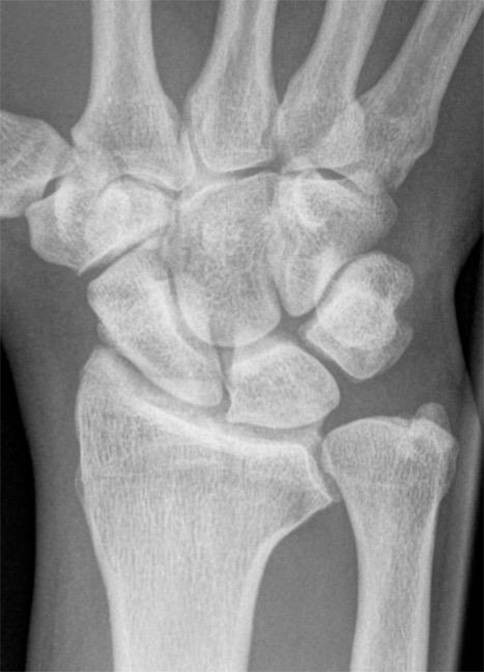

Tolat hat 1992 die DRUG-Konfiguration der Koronarebene in vertikal (Typ I), oblique (Typ II) und revers (umgekehrt schräg, Typ III, Abb. 7) eingeteilt [49]. Die offene **Ulnaverkürzungsosteotomie**

Ulnaverkürzungsosteotomie

birgt bei ungünstiger schräger Konfiguration des DRUGs durch die Veränderung der korrespondierenden Gelenkflächen Risiken für eine Arthrosebildung durch eine Druckerhöhung bei Belastung. Die sichersten Ergebnisse bei der offenen Ulnaverkürzung werden bei einer vertikalen geraden DRUG-Form in der koronaren Ebene erreicht. Bei ungünstiger Konfiguration sollte die Verkürzung nur im Kopfbereich offen metaphysär oder arthroskopisch erfolgen.

**Figure 8:**
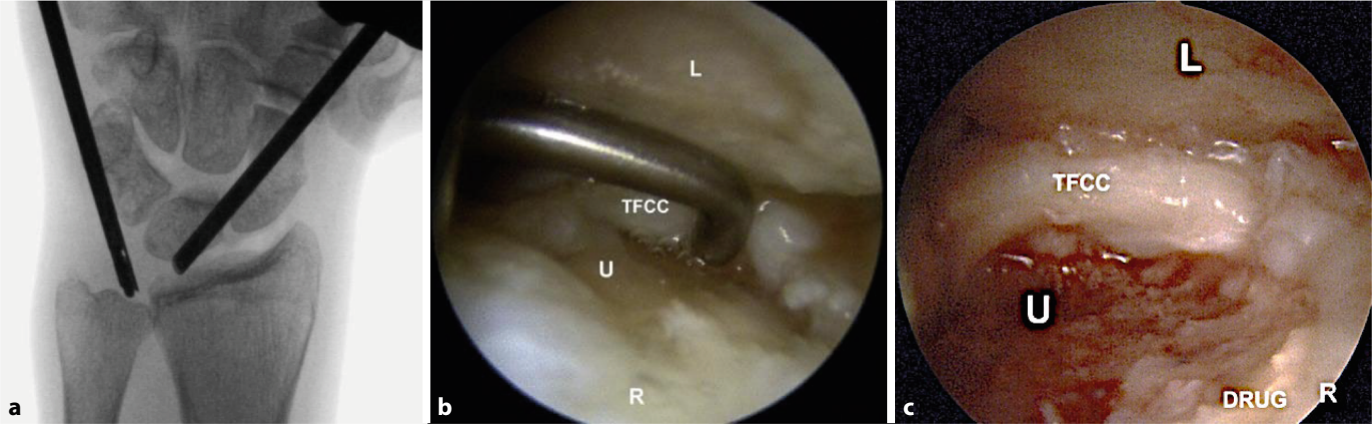

Der Autor bevorzugt bei höhergradigen degenerativen Veränderungen und tendenziell älteren Patienten ein arthroskopisches Vorgehen, das eine schnellere Rekonvaleszenz und weniger Komplikationsmöglichkeit bietet ([50]; Abb. 8).

#### Therapie der Arthrose des distalen radioulnaren Gelenks

Bei einer Arthrose im DRUG kann oft konservativ vorgegangen werden. In einigen Fällen kann eine **arthroskopisch assistierte Resektion**

arthroskopisch assistierte Resektion

von Osteophyten hilfreich sein, wenn diese schmerzhaft blockieren.

Wenn die Beschwerden therapieresistent sind, gab es für lange Zeit nur Resektionen an der distalen Ulna [15]: Bei der **Darrach-Resektion**

Darrach-Resektion

wird des Caput ulnae durch eine horizontale Osteotomie komplett reseziert, und die verbleibende distale Ulna wird lediglich durch einen Muskelkuff geschützt gegen einen Kontakt mit dem distalen Radius [26]. Die Darrach-Operation spielt bei der Rheumachirurgie noch eine Rolle.

Bei der **Hemiresektions-Interpositions-Arthroplastik**

Hemiresektions-Interpositions-Arthroplastik

nach Bowers wird der DRUG-Anteil der Ulna schräg reseziert, wobei der ulnare Teil des Caput ulnae bestehen bleibt. Auch hier wird eine Weichteilinterposition verwendet, um einen Kontakt der Ulna mit dem Radius zu verhindern. Die distalen Ulnaresektionen werden heute nicht mehr breit eingesetzt, haben aber weiterhin ihre Indikationen. Ebenso weitgehend verlassen wurde die Sauvé-Kapandji-Operation, bei der das DRUG fusioniert und an der distalen Ulna ein Segment reseziert wurde. Die Umwendung erfolgte dann proximaler. Dieser Eingriff ermöglichte aber nur erschwert eine Konversion in eine Endoprothesenversorgung.

**Figure 9:**
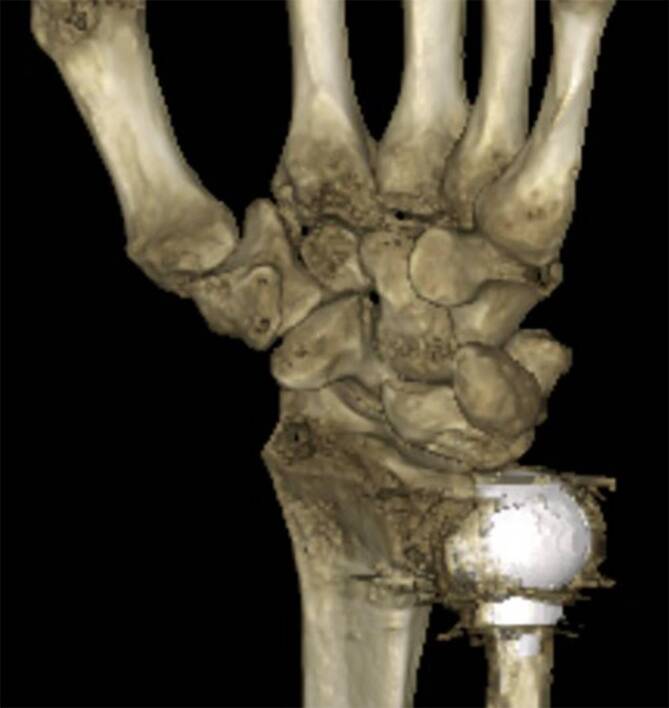

Heute wird bei einer DRUG-Arthrose häufiger als früher schon primär zu einem **endoprothetischen Ulnakopfersatz**

endoprothetischen Ulnakopfersatz

tendiert (Abb. 9). Bei guten Voraussetzungen des Knochenstocks und der ligamentären Voraussetzungen zeigt die Ulnakopfprothese vielversprechende Ergebnisse. Die 10-Jahres-Standzeiten werden mit knapp 90 % angegeben trotz der häufig sekundären Implantation [51, 52].

**Figure 10:**
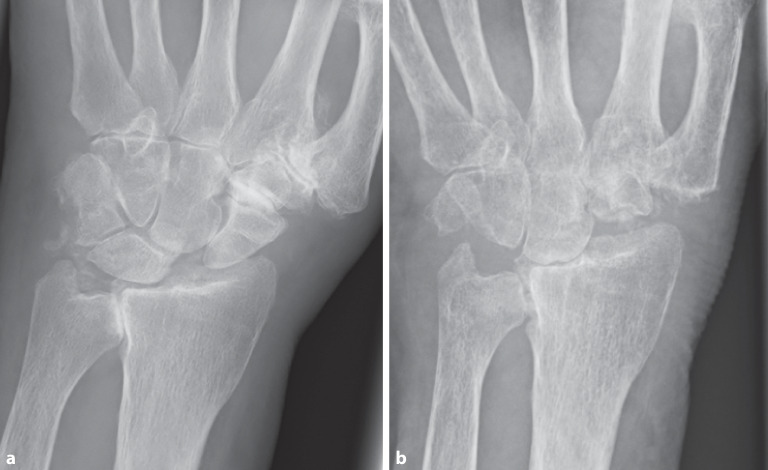

**Figure 11:**
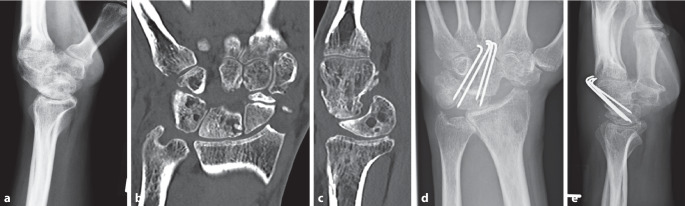

Bei einer kompletten Instabilität im DRUG aufgrund einer Bandinsuffizienz einer Insuffizienz der Membrana interossea (z. B. bei einer Essex-Lopresti-Verletzung) oder einer Radiuskopfresektion mit Längeninkongruenz ist die Implantation einer teilgeführten Prothesenvariante möglich. Diese **Scheker-Prothese**

Scheker-Prothese

ist modular und ersetzt die Funktion des Ulnakopfs, der DRUG-Fläche und des radioulnaren Bandapparates [53].

## Fazit für die Praxis

- Aufgrund des mehrgliedrigen Aufbaus des Handgelenks ist vor einer weiteren Diagnostik eine genaue klinische Untersuchung zur Präzisierung der Fragestellung sinnvoll.
- Ein konventionelles Röntgenbild des Handgelenks ist eine gute Basisdiagnostik, eine Magnetresonanztomographie ist eher für ulnokarpale Beschwerden und Verdacht auf knöcherne Perfusionsstörungen sinnvoll, eine Computertomographie oder digitale Volumentomographie für eine radiokarpale Arthrose zur genaueren Beurteilung vor einer operativen Maßnahme. In Zweifelsfällen kann eine Handgelenkarthroskopie zur Diagnosesicherung erfolgen.
- Konservative Therapiemaßnahmen sollen in anfänglichen Stadien den Vorrang haben, propriozeptives Training ist dabei hilfreich.
- Bei fortgeschrittenen Stadien können sog. Rettungsoperationen wie eine proximale Reihenresektion oder eine mediokarpale Teilarthrodese eine Teilbeweglichkeit erhalten.
- Bei einer Ulnaimpaktion können offene als auch arthroskopische Verfahren zur Ulnaverkürzung eingesetzt werden.

## CME-Fragebogen

Welche Handwurzelknochen sind bei der radiokarpalen Arthrose betroffen?

Triquetrum, Hamatum und Capitatum

Capitatum und Hamatum

Capitatum, Trapezium und Trapezoideum

Lunatum und Skaphoid

Skaphoid und Trapezium

Welcher Handwurzelknochen ist bei der Ulnaimpaktion typischerweise betroffen?

Hamatum

Skaphoid

Pisiforme

Capitatum

Lunatum

Eine 68-jährige Patientin stellt sich vor mit Beschwerden bei der Pro- und Supination im Handgelenk. Tennisspielen wird zunehmend schmerzhaft. Das Abstützen auf die Faust ist wenig eingeschränkt. Bei der Abstützung auf das extendierte Handgelenk bestehen ulnokarpale Beschwerden. Welche primäre diagnostische Abklärung halten Sie für zielführend?

CT(Computertomographie)-Handgelenk

Sonographie DRUG (distales radioulnares Gelenk)

Projektionsradiographie p.a. (posterior anterior), seitlich und Faustschlussaufnahme

MR-(Magnetresonanz)-Arthrographie Handgelenk

Computertomographie-Arthrographie Handgelenk

Ein 73-jähriger Patient ist seit längerer Zeit mit einer Rhizarthrose in Behandlung. Eine Röntgendiagnostik liegt nicht vor. Welche Arthrose kann aufgrund der anatomischen Nähe ähnliche Beschwerden wie die Rhizarthrose auslösen oder zusätzlich zur Rhizarthrose am Handgelenk vorkommen?

DRUG(distales radioulnares Gelenk)-Arthrose

SLAC(„scapholunate advanced collapse“)-Wrist 2°

SCAC(„scaphoid chondrocalcinosis advanced collapse“)-Wrist

STT(skaphotrapezotrapezoidal)-Arthrose

Lunatumnekrose Stadium IV

Beschreiben Sie die Veränderung der Skaphoidstellung auch in der Projektionsradiographie bei einer höhergradigen SL(skapholunär)-Insuffizienz, die zu einer SLAC(„scapholunate advanced collapse“)-Wrist führt.

Das Skaphoid dreht sich rotierend in Richtung des Lunatums und subluxiert palmar.

Aufgrund der DISI(„dorsally intercalated segment instability“)-Stellung im Lunatum bewegt sich das Skaphoid ebenfalls in die Extension.

Das Skaphoid flektiert, wandert nach dorsal und drückt auf die dorsale Radiuslippe.

Die Durchblutung des Skaphoids verschlechtert sich, und es kommt zur Infraktion.

Das Skaphoid erzeugt im STT(skaphotrapezotrapezoidal)-Gelenk eine Schleifarthrose.

Fünfzehn Jahre nach einer nicht diagnostizierten Skaphoidfraktur im mittleren Drittel zeigt eine Dünnschichtcomputertomographie eine Humpback-Deformität am Skaphoid, eine Pseudarthrose mit Zystenbildung und eine Gelenkspaltaufhebung in der Fossa scaphoidea radial der Pseudarthrose und einen spitz wirkenden Processus styloideus radii. Zwischen dem dorsal gestellten Lunatum und dem Capitatum fällt dorsal eine Gelenkspaltreduktion auf. Welche Diagnose erscheint naheliegend?

SLAC(„scapholunate advanced collapse“)-Wrist 2°

SCAC(„scaphoid chondrocalcinosis advanced collapse“)-Wrist 1°

SNAC(„scaphoid nonunion advanced collapse“)-Wrist 3°

SLAC(„scapholunate advanced collapse“)-Wrist 3°

SNAC(„scaphoid nonunion advanced collapse“)-Wrist 2°

Welche operative Möglichkeit bietet sich bei einer symptomatischen hochgradigen radiokarpalen Arthrose nach Radiusfraktur an, wenn mediokarpal keine Arthrosezeichen festzustellen sind?

Mediokarpale Teilarthrodese

Radioskapholunäre Fusion

Proximale Reihenresektion

Arthroskopische Resektionsarthroplastik im STT(skaphotrapezotrapezoidal)-Gelenk

Implantation einer Ulnakopfprothese

Welche operative Option kann bei der ulnokarpalen Arthrose aufgrund eines Ulnaimpaktionssyndroms überlegt werden?

Mediokarpale Teilarthrodese

STT(skaphotrapezotrapezoidal)-Arthrodese

Ulnakopfresektion (Darrach-Operation)

Arthroskopische Ulnakopfteilresektion (Wafer-Prozedur)

Hemiresektions-Interpositions-Arthroplastik nach Bowers

Welche Handwurzelknochen werden bei der proximalen Reihenresektion (PRC) entfernt?

Skaphoid, Triquetrum, Lunatum

Skaphoid, Trapezium, Trapezoideum

Triquetrum, Hamatum, Lunatum

Trapezium, Capitatum, Lunatum

Triquetrum, Skaphoid, Hamatum

Welche Handwurzelknochen werden bei einer mediokarpalen Teilresektion (MKTA) fusioniert?

Skaphoid, Trapezium, Trapezoideum

Lunatum, Skaphoid, Capitatum

Capitatum, Lunatum, Hamatum, Triquetrum

Skaphoid, Capitatum, Lunatum, Hamatum

Capitatum, Skaphoid, Trapezium, Trapezoideum
